# Supplementary material for: Improved analyses of GWAS summary statistics by reducing data heterogeneity and errors
Source: Nat Commun. 2021 Dec 8;12:7117. doi: 10.1038/s41467-021-27438-7 (PMC8654883; doi:10.1038/s41467-021-27438-7)
Supplement: Supplementary file 1 — Supplementary information [file 41467_2021_27438_MOESM1_ESM.pdf]

Improved analyses of GWAS summary statistics by reducing data heterogeneity and errors

Chen et al.

Supplementary Figures 1-15

Supplementary Tables 1-14

Supplementary Notes

Supplementary References

Supplementary Figures

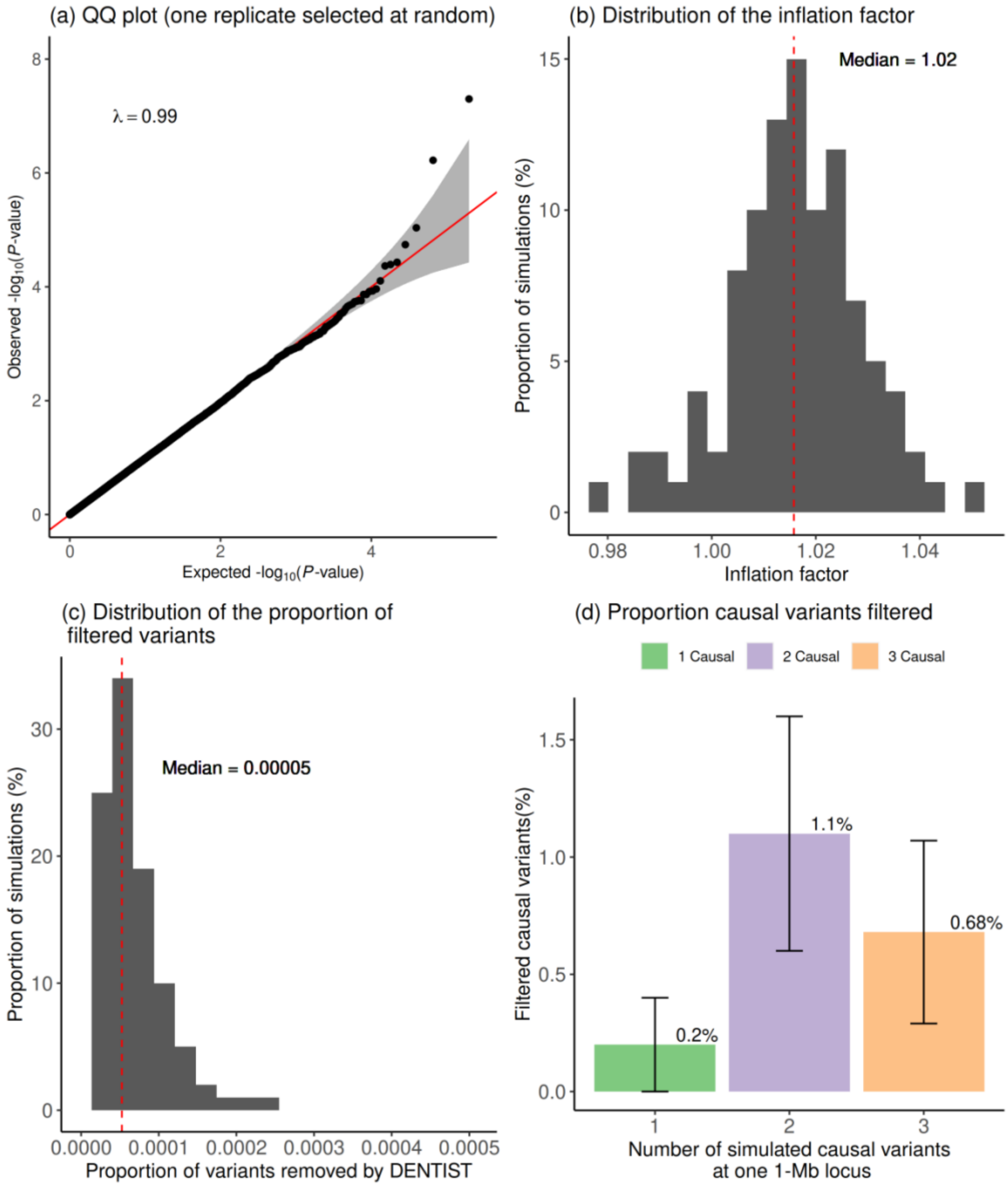

Supplementary Figure 1. Statistical properties of the DENTIST test statistic in the absence of errors. We simulated a quantitative trait with 50 causal variants selected at random using the UK10K-WGS data without introducing genotyping or allelic errors. The inflation factor (λ) is defined as the median of the DENTIST test statistics of all the variants divided by 0.455, i.e., the expected median of χ_1^2 under the null. The DENTIST test *P*-value was calculated from a one-sided χ_1^2 test. The QQ plot of a simulation replicate selected at random is shown in panel a). The gray shade area represents the 95% confidence interval. Shown in panels b) and c) are the distributions of the inflation factor and the proportion of variants removed by DENTIST, respectively, from 100 simulation replicates, with the median value marked by a red vertical dashed line. In panel d), we

performed additional simulations with one, two, or three causal variants ($r^2 < 0.1$ between the causal variants and $q^2 = 2\%$ for each causal variant) at a 1-Mb locus using the same data. Each scenario was repeated 440 times with the causal variants resampled in each replicate. Shown on the y-axis is the proportion of causal variants filtered out by DENTIST across simulation replicates pooled across all the causal variants. The error bar represents +/- one s.e. of the estimated proportion. Source data are provided as a Source Data file.

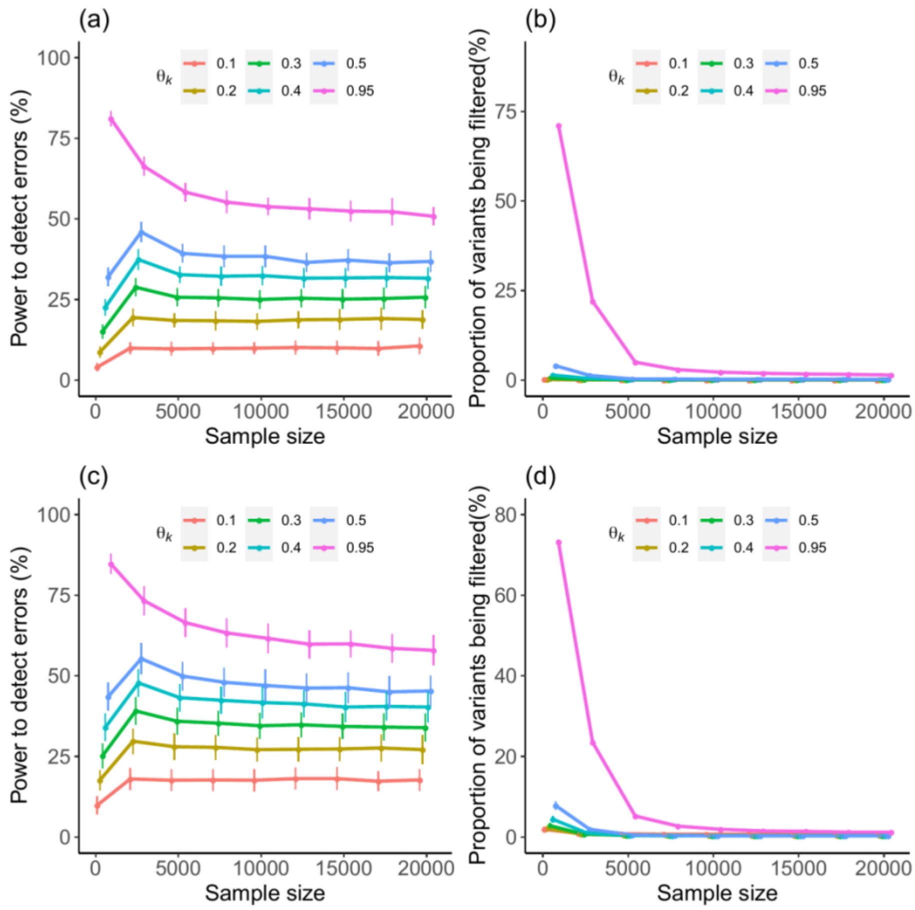

Supplementary Figure 2. The choice of the SVD parameter θ_k and the reference sample size. To investigate the effects of the SVD parameter θ_k (see Methods for the definition of θ_k) and reference sample size on the performance of DENTIST, we analyzed the GWAS summary data simulated based on UK10K-WGS (n = 3,642) as described in the main text (simulating genotyping and allelic errors on 0.5% of the variants, respectively, on chromosome 22). UKBv3-20K (20,000 unrelated individuals from the UKB) was used as the LD reference for DENTIST analysis. Power is defined as the number of correctly identified erroneous variants by DENTIST divided by the total number of simulated erroneous variants. We repeated the analysis using a subset of the UKBv-20K data (n_{ref} = 500, 2,500, 5,000, 7,500, 10,000, 12,500, 15,000 or 17,500) with different levels of θ_k ($\theta_k = 0.1, 0.2, 0.3, 0.4, 0.5$ or 1). The results shown in panels a) and b) suggest the use $\theta_k = 0.5$ and n_{ref} > 5,000 in practice. We further performed simulations based on the genotype data of 20,000 unrelated UKB individuals (independent of UKBv3-20K) using the same strategy as in the UK10K simulation and using reference LD calculated from each subset of the UKBv-20K data (n_{ref} = 500, 2,500, 5,000, 7,500, 10,000, 12,500, 15,000 or 17,500). The results shown in panels c) and d) suggest that the choice of θ_k and minimal n_{ref} do not seem to depend on discovery GWAS sample size. In panels a) and c), each error bar represents +/- one standard error of the corresponding estimate computed from 100 simulation replicates. Source data are provided as a Source Data file.

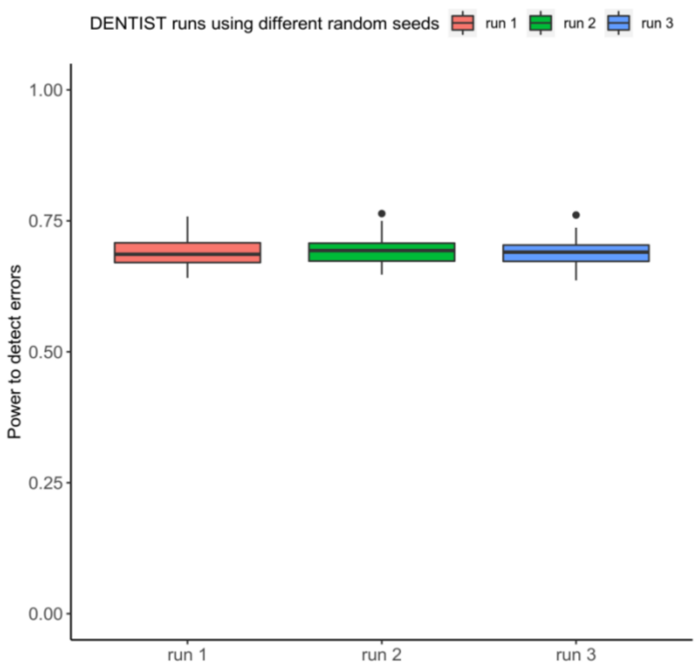

Supplementary Figure 3. The stability of DENTIST to random seed. We quantify how random seed used in DENTIST can affect the detection of errors by apply 3 DENTIST runs with different random seeds to GWAS summary data simulated based on UK10K-WGS (n = 3,642) as described in the main text (simulating genotyping and allelic errors on 0.5% of the variants, respectively, on chromosome 22). The power is quantified as the proportion of both errors detected from DENTIST based on 100 replicates. Each box plot depicts median, 25th and 75th percentiles (interquartile range, IQR), and whiskers that extend to maximum and minimum values. Source data are provided as a Source Data file.

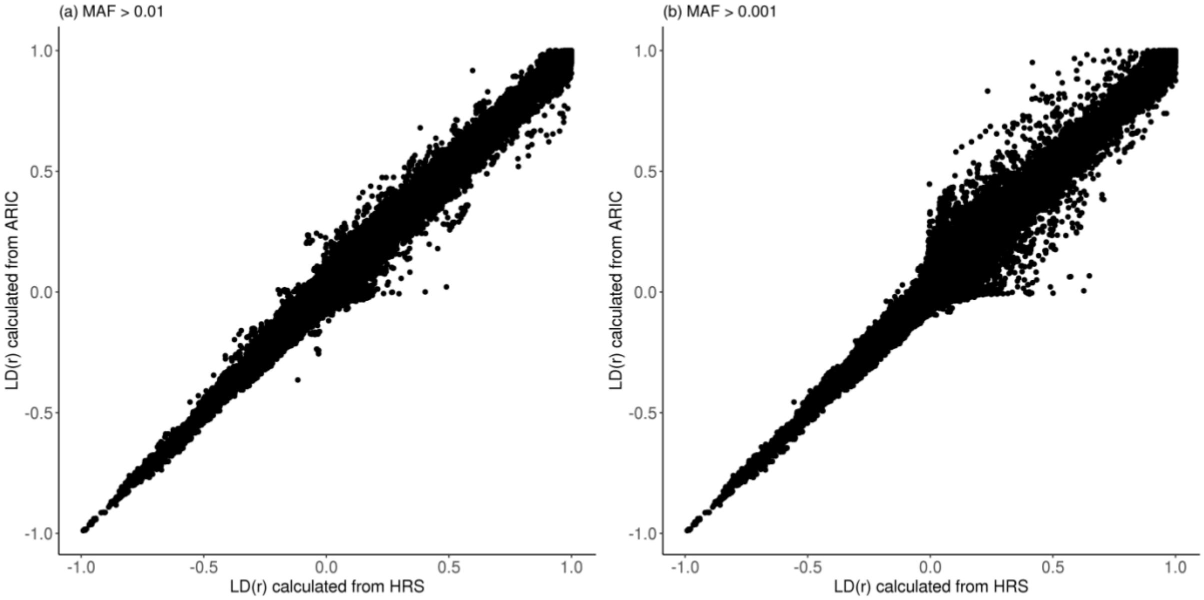

Supplementary Figure 4. Comparison of LD between two samples of European ancestry. Shown are LD correlations (r) between pairwise variants at a randomly selected locus on chromosome 22 computed from ARIC plotted against those from HRS. Panel a): variants with MAF >0.01. Panel b): variants with MAF >0.001. Source data are provided as a Source Data file.

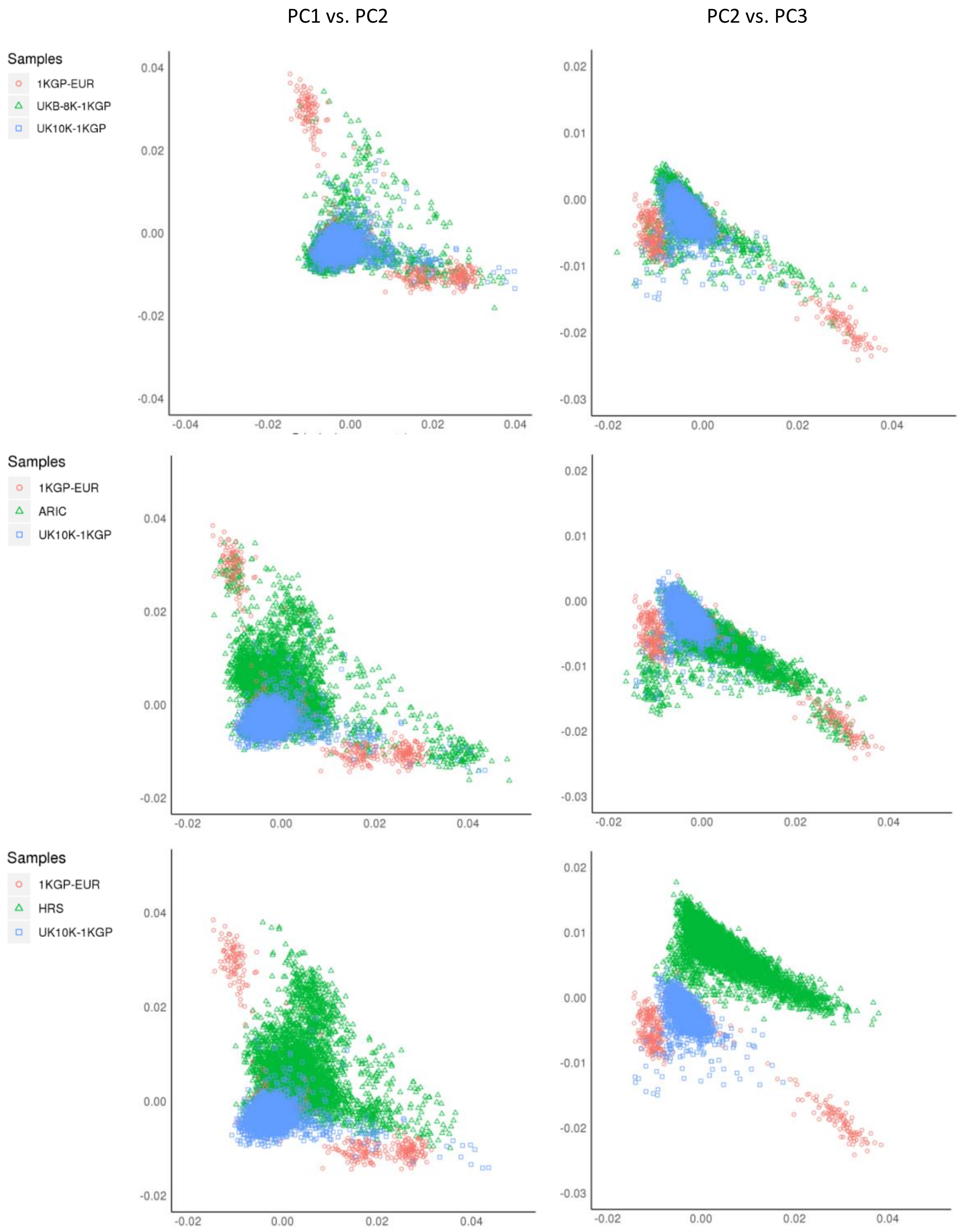

Supplementary Figure 5. Principal component analysis of the LD reference samples. We combined the genotype data of HRS, ARIC and UKB-8K-1KGP with those of 1KGP-EUR and performed a principal component analysis (PCA) using the variants with MAF>0.05. The panels on the left-hand side show the plots of PC1 against PC2, and those on the right-hand side show the plots of PC3 against PC2. As expected, UKB-8K-1KGP shows a greater overlap with UK10K-1KGP than ARC or HRS. Source data are provided as a Source Data file.

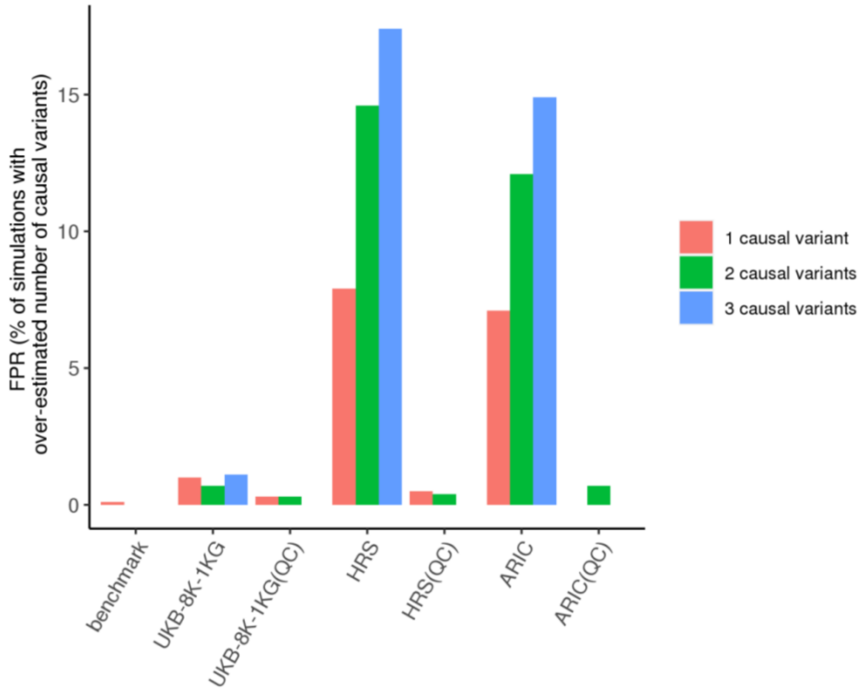

Supplementary Figure 6. FPR of COJO with and without DENTIST QC in simulations with one, two or three causal variant(s) at one locus. Based on simulations with 1, 2 or 3 causal variant(s), we assessed the FPRs of COJO when performed with and without DENTIST-based QC (FPR is defined as the proportion of simulations in which the number of COJO signals is larger than the number of causal variants). The x-axis labels indicate the LD reference samples, and those performed after DENTIST QC are labeled with “QC” in the parentheses. Source data are provided as a Source Data file.

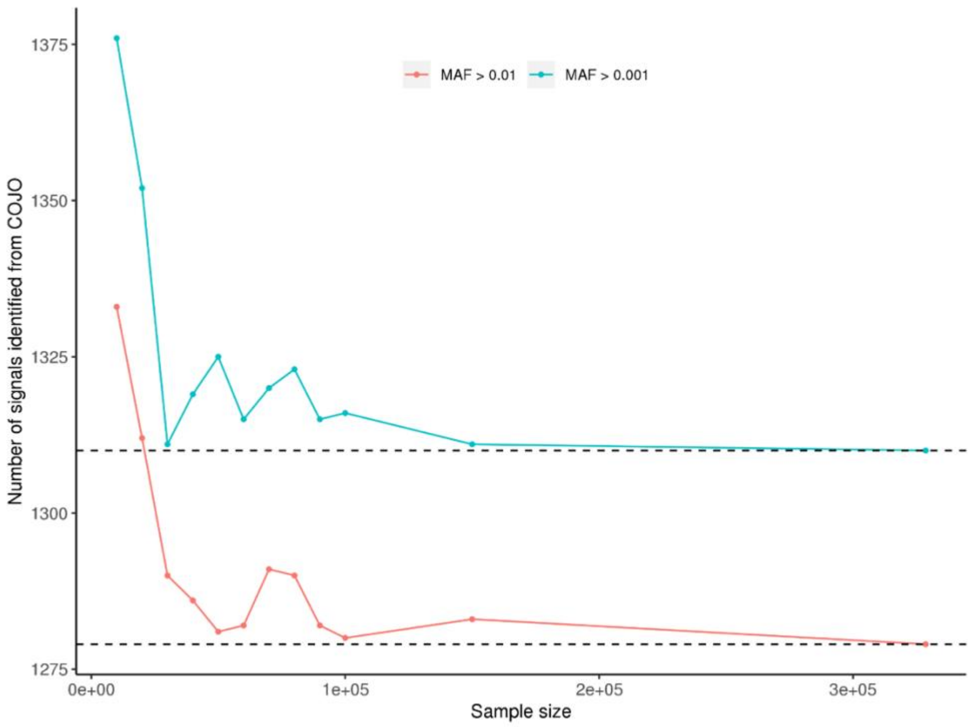

Supplementary Figure 7. The change of the number COJO signals with the increase of the in-sample LD reference sample size. By subsetting the discovery GWAS sample (328,577 unrelated UKB participants), we created 11 in-sample LD references with sample sizes ranging from 10,000 to 150,000. As the reference sample size (n_{ref}) increases, the number of signals identified from COJO with MAF>0.01 first decreases sharply from 1,333 at n_{ref} =10,000 to 1,290 at n_{ref} =30,000, before stabilizing in a range between 1,280 and 1,291, close to the benchmark of 1,279 signals identified using the whole discovery GWAS sample as the reference (the right-most dot on the plot). The COJO results for variants with MAF > 0.001 show a similar pattern, i.e., the number of COJO signals deceases sharply when n_{ref} increases from 10,000 to 30,000 and stabilizes in the range between 1,310 and 1,325. Source data are provided as a Source Data file.

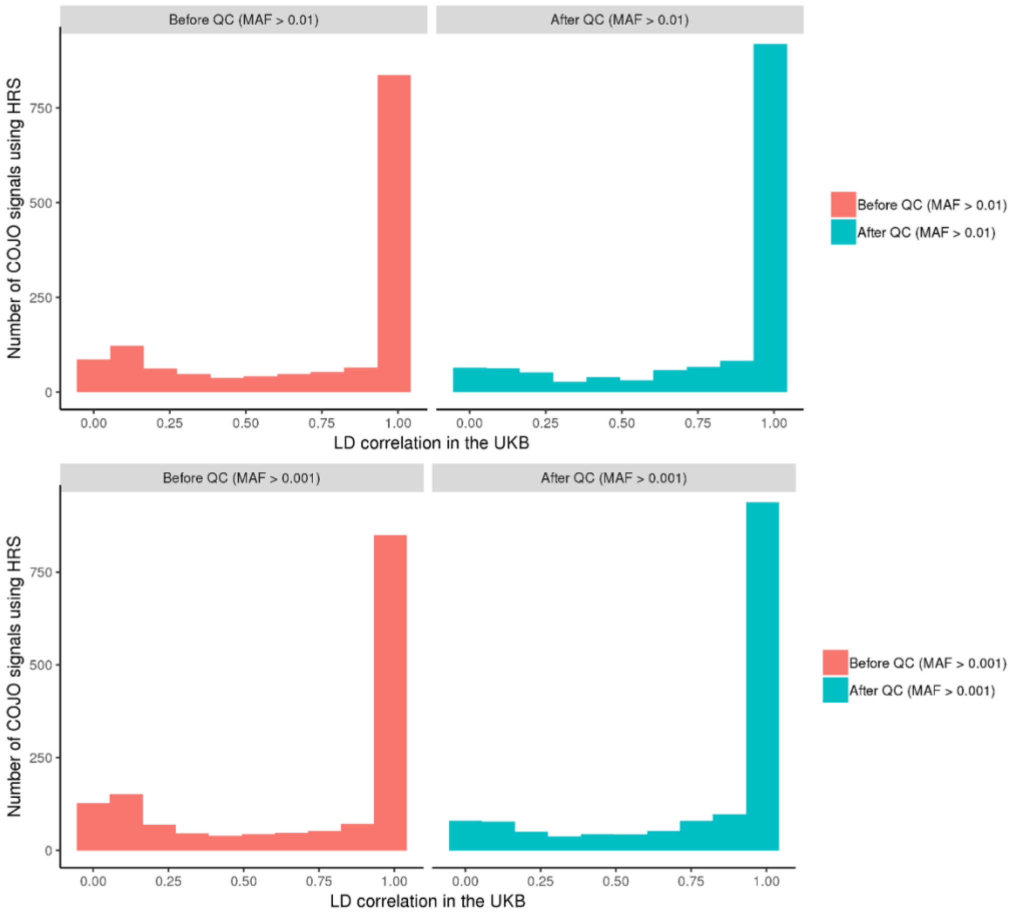

Supplementary Figure 8. LD correlations between the COJO signals identified using HRS as the reference and their best matching signals identified from the benchmark analysis. Shown are the LD correlations between the COJO signals for height, identified using HRS as the LD reference before and after DENTIST-based QC, and their best matching signals identified from the benchmark analysis using the whole discovery GWAS (UKBv3-329K) as the LD reference. For variants with MAF >0.01, a few signals identified using HRS shown on the top-right panel show weak LD (r^2 < 0.3) with the benchmark signals. After DENTIST-QC, the number of poorly correlated signals is greatly reduced as shown on the top-right panel (r^2 < 0.3). On the other hand, the DENTIST-based QC leads to more strong correlations (r^2 > 0.9). A similar pattern is observed for variants with MAF>0.001 (shown in the two bottom panels). Source data are provided as a Source Data file.

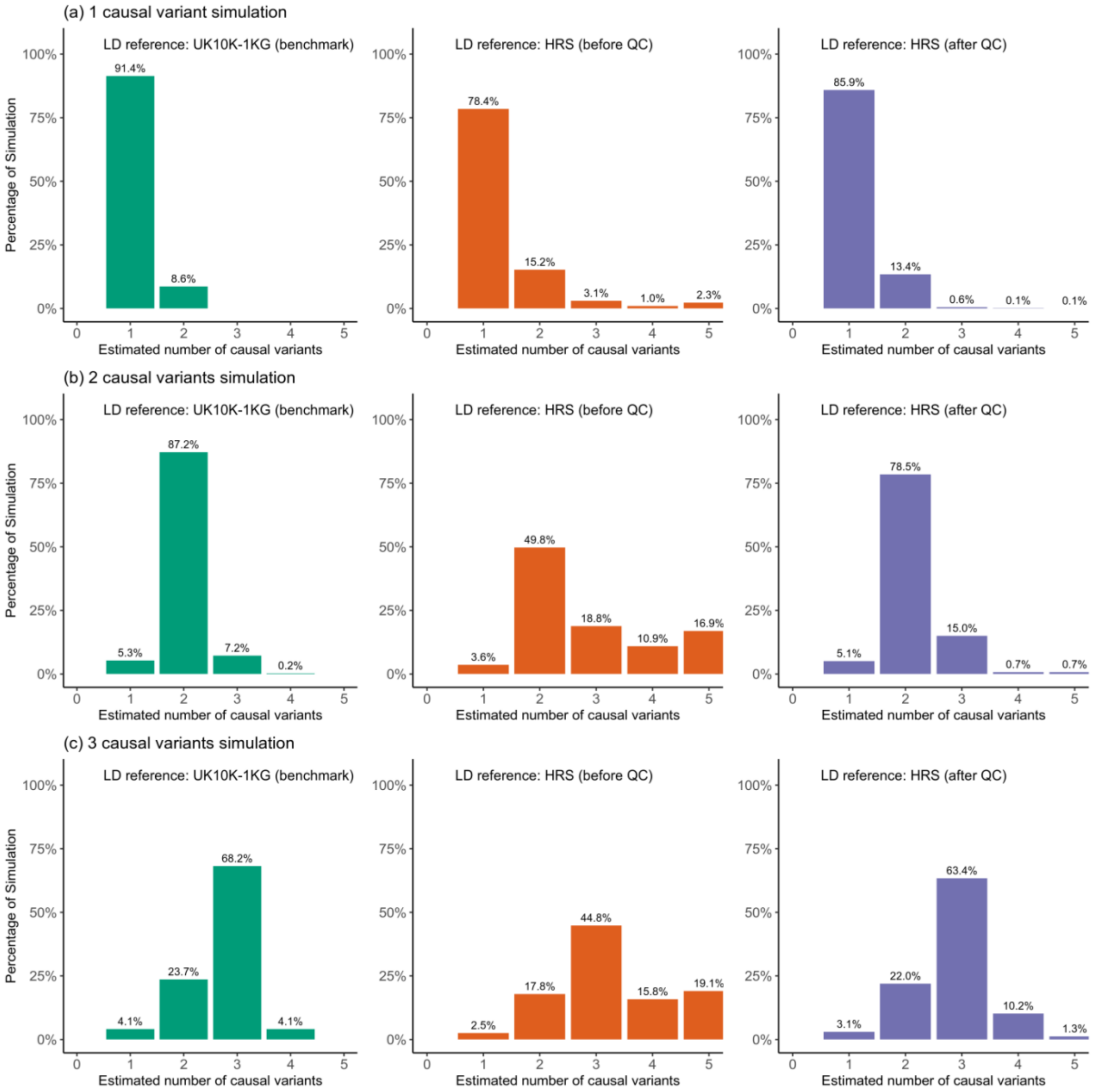

Supplementary Figure 9. FINEMAP analysis using out-of-sample LD from HRS with or without DENTIST QC compared to the benchmark. We assessed the performance of FINEMAP by comparing the estimated number of causal variants to the true number of causal variants in simulations with 1, 2 or 3 causal variants at on one locus. Each scenario was repeated 200 times with the causal variants resampled in each replicate. Plotted is the proportion of simulation replicates in which the estimated number of causal variants from FINEMAP was consistent with the true number. Benchmark: FINEMAP analysis using in-sample LD (without DENTIST QC). Source data are provided as a Source Data file.

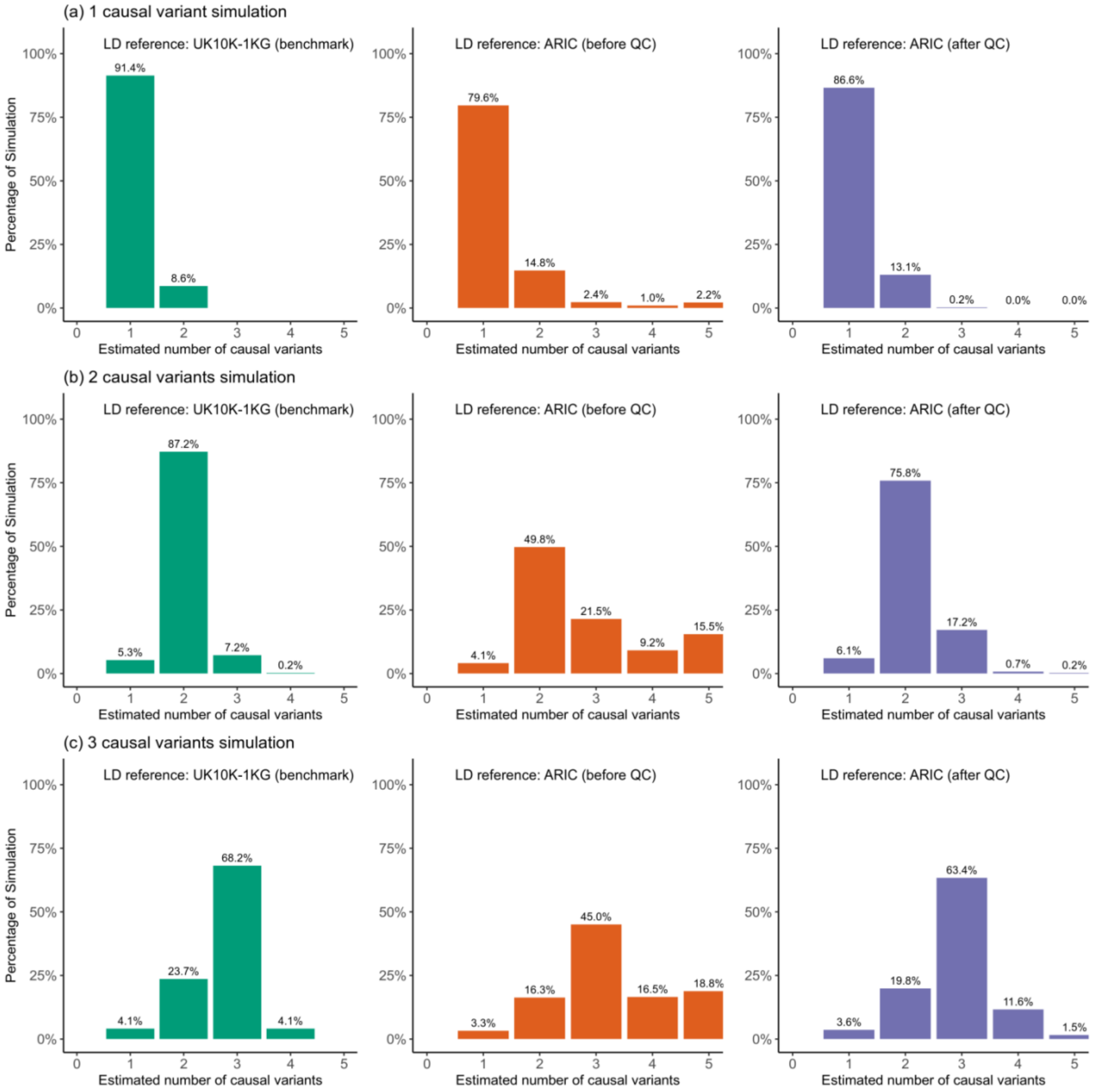

Supplementary Figure 10. FINEMAP analysis using out-of-sample LD from ARIC with or without DENTIST QC compared to the benchmark. We assessed the performance of FINEMAP by comparing the estimated number of causal variants to the true number of causal variants in simulations with 1, 2 or 3 causal variants at on one locus. Each scenario was repeated 200 times with the causal variants resampled in each replicate. Plotted is the proportion of simulation replicates in which the estimated number of causal variants from FINEMAP was consistent with the true number. Benchmark: FINEMAP analysis using in-sample LD (without DENTIST QC). Source data are provided as a Source Data file.

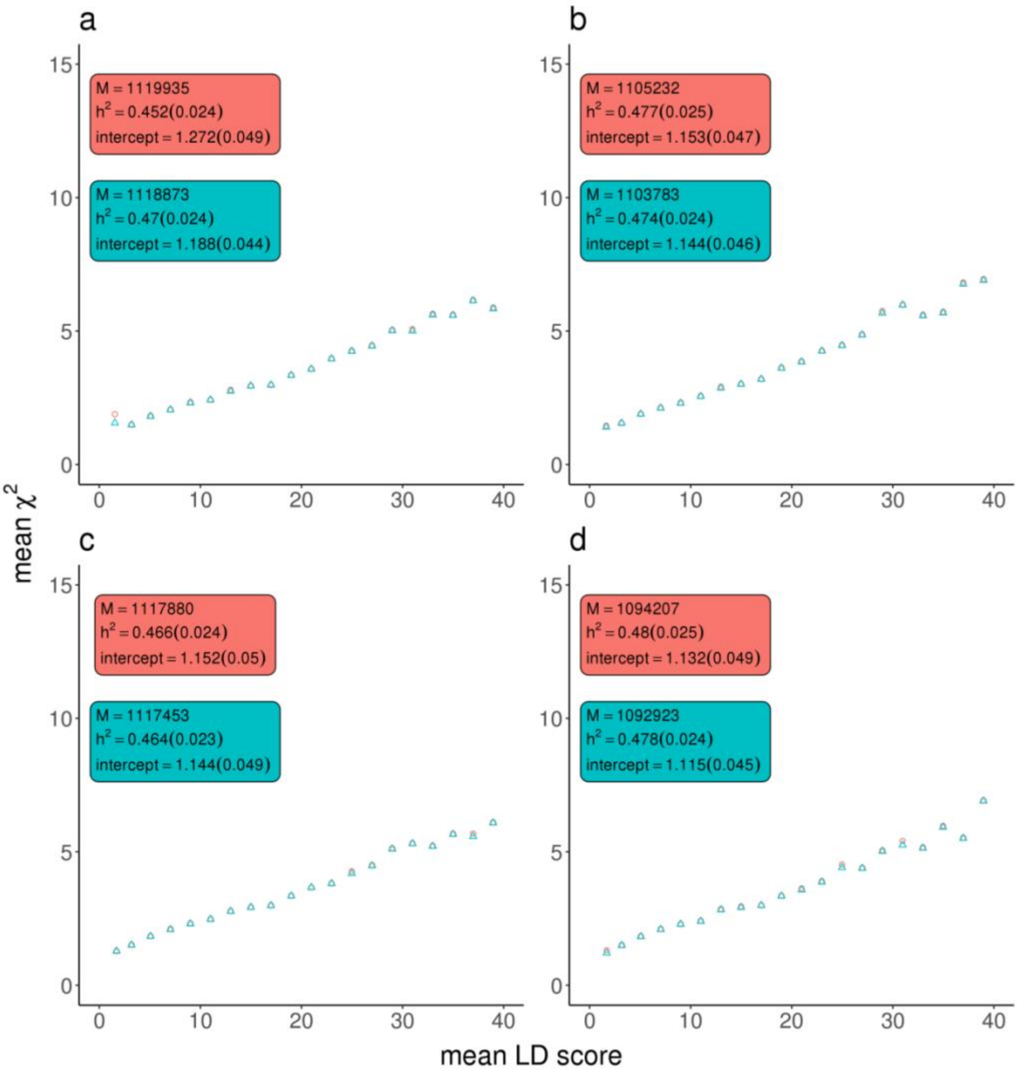

Supplementary Figure 11. The effect of DENTIST-based QC on LDSC analysis of the UKB height summary data. We assessed the effect of DENTIST on LDSC when different LD references were used, including **a**) HRS, **b**) ARIC, **c**) UKB-8K-1KGP, and **d**) UK10K-WGS. For each reference sample, LDSC was performed before and after DENTIST-based QC, and the corresponding results are shown in the red and cyan text boxes, respectively, on each plot. The variants are binned by their LD scores. Each dot on the plots represents the mean LD score value of each bin on the x-axis and the mean χ^2 value on the y-axis, with those before and after DENTIST-based QC in red and cyan colors respectively. In the textbox, "M" represents the number of variants, "h^2_{SNP}" represents the estimate of SNP-based heritability, and "intercept" represents the LDSC intercept, with the corresponding standard errors given the parentheses. Source data are provided as a Source Data file.

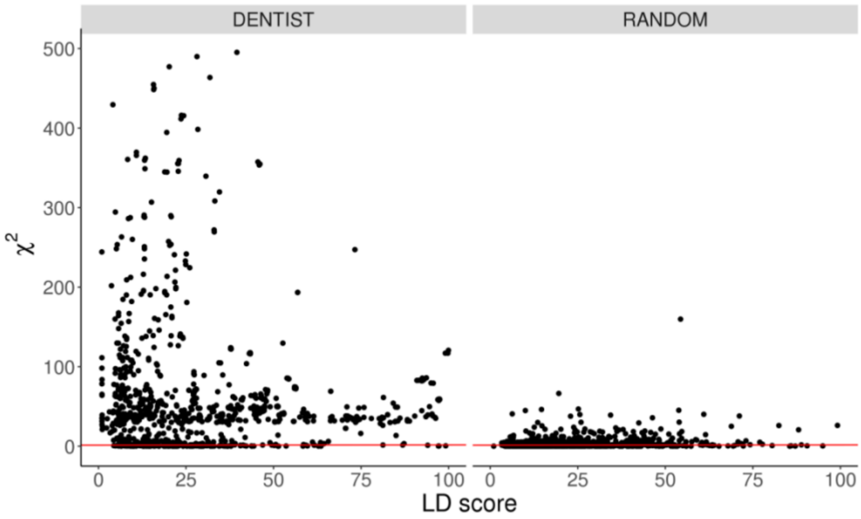

Supplementary Figure 12. A plot of LD scores of the variants detected by DENTIST against their GWAS test-statistics for height in the UKB. This plot shows the LD scores and χ^2 statistics of variants identified from a DENTIST analysis of the UKB height GWAS data (n = 328,577 unrelated UKB participants) using HRS as the LD reference compared to variants selected at random. The red line represents the regression line with its intercept and slope estimated from the LDSC analysis of all the variants. Source data are provided as a Source Data file.

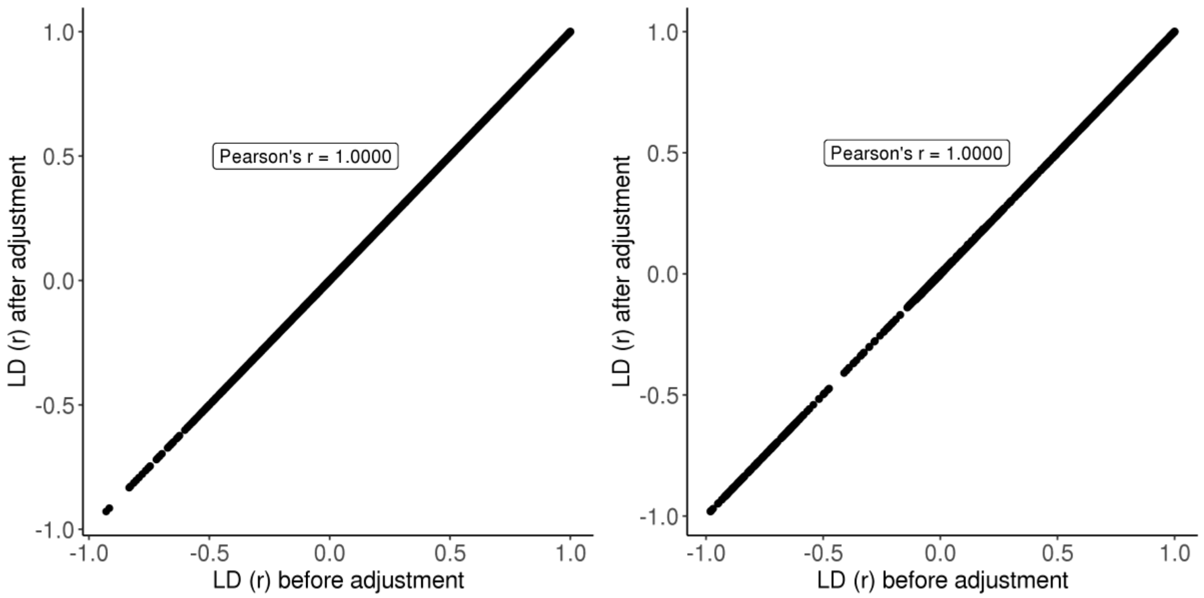

Supplementary Figure 13. Correlation between LD correlation values before and after adjusting genotypes for genetic PCs. We computed the LD correlation (r) values between pairwise common variants (MAF > 0.01) on chromosome 22 using the UK10K genotype data before and after adjusted for the first two genetic PCs derived from genome-wide common variants. Shown in the left panel is the comparison of LD values calculated between all pairwise variants less than 1Mb away from each other, and the right panel shows the comparison restricted to LD between variants that are significantly associated with one of the two PCs (association analysis P-value < 0.01, calculated from a one-sided χ_1^2 test.). Source data are provided as a Source Data file.

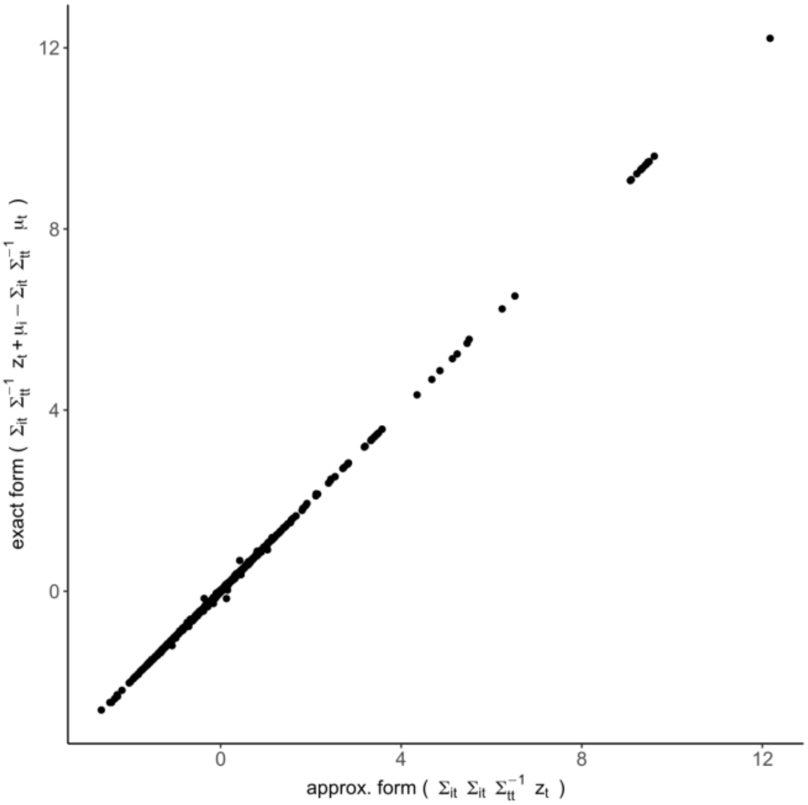

Supplementary Figure 14. Comparison between imputed z-score derived under null model (approx. form) and imputation under fixed-effect model (exact form). We used UK10K.WGS sample to simulate a phenotype caused by a variant *c* with effect denoted by λ_c and heritability fixed to $h^2 = 0.05$. Therefore, the expected values for each marginal effect measured in z-score, [illegible], equals to $\Sigma_{ic}\lambda_c$, where Σ_{ic} is the LD correlation between variants *i* and *c*. Source data are provided as a Source Data file.

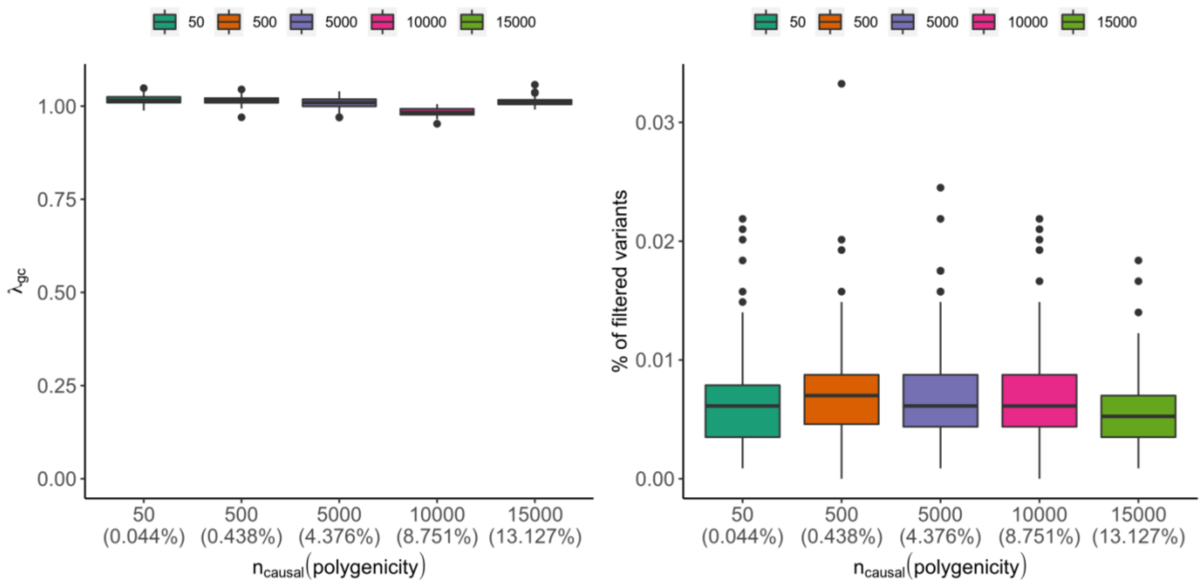

Supplementary Figure 15. The impact of the genetic architecture of the trait on DENTIST. We assessed DENTIST under a range of MAF- and LD-dependent genetic architectures by simulation using data on chromosome 22 from UK10K-WGS, ranging from oligogenic (m_{causal} = 50) to very polygenic ones (m_{causal} =15,000, accounting for 13.1% of all the variants). The effects were sampled from $N(0, [2p(1-p)]^{-1})$ with p being the MAF, and the trait heritability h^2 was set to 20%. The causal variants were sampled from a set of variants with larger LD scores than average, to generate a LD-dependent genetic architecture. Each scenario was repeated 100 times, with the causal variants and their effect sizes resampled in each replicate. No errors were simulated in this scenario. Shown in panels a) and b) are the inflation factor of the DENTIST test statistic and the proportion of variants removed by DENTIST, respectively. This is no relationship between the inflation factor of DENTIST test statistic (or the proportion of variants filtered by DENTIST) and the number of causal variants simulated, and all these results are similar to those from simulations based on a simple genetic architecture as presented in Supplementary Figure 1, suggesting the robustness of DENTIST to the genetic architecture of the trait. Each box plot depicts median, 25th and 75th percentiles (interquartile range, IQR), and whiskers that extend to maximum and minimum values. Source data are provided as a Source Data file.

Supplementary Tables

Supplementary Table 1. Proportion of simulated erroneous variants identified by standard QC.

LD reference	% variants with allelic errors detected by the ΔAF test			% variants with genotyping errors detected by the HWE test
	MAF ≥ 0.01	MAF > 0.4	MAF > 0.45	
UKB-8K-1KGP	95±1.0	57±6.8	16±6.9	45±2.1
UK10K–WGS	94±1.1	50±6.6	/	52±2.3

Shown are mean ± standard error.

Supplementary Table 2. Power in detecting simulated genotyping and allelic errors in UK10K-WGS using out-of-sample LD from UKB-8K-1KGP.

	**Variants stratified into bins by |z-score| or p-value**						**All**
	|z-score| [0, 1)	|z-score| [1, 2)	|z-score| [2, 3)	|z-score| [3, 4)	|z-score| >4	*P*-value $<5\times10^{-8}$	
Allelic error	57.4±1.2	99.2±0.3	100±0.0	100±0.0	100±0.0	100±0.0	73.4±0.9
5% genotyping error rate	18.4±0.5	21.8±0.7	30.7±1.7	49.2±4.4	56.9±5.8	84.8±6.2	20.9±0.4
10% genotyping error rate	26.2±0.6	31.4±1.0	47.6±2.2	61.3±5.4	92.3±3.7	100±0.0	29.9±0.5
15% genotyping error rate	29.8±0.7	38.7±1.2	52.1±2.6	84.7±3.9	92.3±5.2	100±0.0	34.8±0.6
All genotyping errors	23.7±0.3	29.0±0.5	40.9±1.2	62.8±2.8	75.3±3.5	93.5±2.8	27.2±0.3

"All genotyping errors" refers to all the genotyping errors regardless of genotyping error rate setting. Shown are mean ± standard error.

Supplementary Table 3. Proportion of variants removed by different QC strategies in the simulations based on the UK10K-WGS data.

Reference sample	Standard QC	DENTIST-based QC
**Among all the variants**		
UKB-8K-1KGP	0.6 ± 0.01%	0.20±0.02%
UK10K-WGS	0.7 ± 0.01%	0.13±0.01%
**Among the causal variants**		
UKB-8K-1KGP	0.44 ± 1.1%	0.99±1.4%
UK10K-WGS	0.44 ± 1.1%	0.19±0.63%

Supplementary Table 4. Fold enrichment in probability of detecting an erroneous variant using DENTIST before and after standard QCs.

	**Allelic errors**	**Genotyping errors**			
		Genotyping error rate = 5%	Genotyping error rate = 15%	Genotyping error rate = 15%	All
**Using UKB-8K-1KGP as the reference (out-of-sample LD)**					
DENTIST before standard QCs	82 ± 4.7	33 ± 4.9	44 ± 6.3	49 ± 5.8	42 ± 3.9
DENTIST after standard QCs	366±58.7	105±21.8	150±26.4	176±34.4	137±17.0
**Using UK10K-WGS as the reference (in-sample LD)**					
DENTIST before standard QCs	103 ± 4.0	51 ± 5.0	64 ± 5.9	69 ± 5.4	61 ± 3.0
DENTIST after standard QCs	575±87.8	325±51.9	212±32.8	286±40.2	261±27.7

"All" refers to all the genotyping errors regardless of the genotyping error rate setting. Shown are mean ± standard error.

Supplementary Table 5. Power of DENTIST in detecting simulated genotyping and allelic errors using the UK10K-WGS sample as the LD reference.

	**|z-score| [0, 1)**	**|z-score| [1, 2)**	**|z-score| [2, 3)**	**|z-score| [3, 4)**	**|z-score| >4**	**|z-score| >5.45**	**|z-score| >0**
**Allelic errors**	59.8±1.1	98.3±0.4	100.0±0.0	100.0±0.0	100.0±0.0	100.0±0.0	74.6±0.8
**Genotyping errors (5%)**	24.8±0.5	29.7±0.7	35.7±1.6	53.5±4.0	64.3±5.2	81.8±5.8	27.6±0.4
**Genotyping errors (10%)**	33.9±0.6	39.6±0.9	53.2±2.0	77.9±4.1	96.7±2.3	97.0±3.0	37.7±0.5
**Genotyping errors (15%)**	37.2±0.7	45.3±1.1	58.9±2.4	79.4±4.1	96.9±3.1	100.0±0.0	41.7±0.6
**All genotyping errors**	30.6±0.3	36.6±0.5	46.3±1.1	67.6±2.5	81.3±2.9	91.0±2.9	34.1±0.3

“All genotyping errors” refer to all the genotyping errors regardless of the genotyping error rate setting. |z-score| >5.45 corresponds to P-value $<5\times10^{-8}$. Shown are mean ± standard error.

Supplementary Table 6. The loss of power in GWAS due to imperfect tagging of the causal variants.

	**Common**	**Rare**
**Variant tagging (absolute value of Pearson's correlation between imputed and sequenced genotypes)**	0.77± 0.162	0.46±0.257
**Proportion of simulations containing ≥1 genome-wide significant signals**	89%	50%

Shown are mean ± standard error.

Supplementary Table 7. COJO analysis of the UKB GWAS summary data for height using the whole discovery or a subset of the discovery sample as the LD reference.

Reference sample size	MAF > 0.01	MAF > 0.001
10000	1,333	1,376
20000	1,312	1,352
30000	1,290	1,311
40000	1,286	1,319
50000	1,281	1,325
60000	1,282	1,315
70000	1,291	1,320
80000	1,290	1,322
90000	1,282	1,315
100000	1,280	1,316
150000	1,283	1,311
All (n=328577)	1,279	1,310

Supplementary Table 8. The accuracy (r^2) of using the COJO signals identified from the UKB to predict height in HRS.

	LD reference for COJO and DENTIST		
	**ARIC**	**UKBv3-8K**	**UKBv3-20K**
Using COJO signals at MAF > 0.01			
COJO only	0.18±0.007	0.19±0.007	0.19±0.007
COJO + DENTIST	0.19±0.007	0.19±0.007	0.19±0.007
Using COJO signals at MAF > 0.001			
COJO only	0.18±0.007	0.19±0.007	0.19±0.008
COJO + DENTIST	0.19±0.007	0.19±0.007	0.19±0.007

Note: we constructed a polygenic score for height in HRS based on the COJO signals identified from the UKB height data using three different references. The numbers of COJO signals identified using different LD references before and after DENTIST can be found in Table 3. Shown in this table are mean ± standard error of the correlation squared between the PRS and the real height phenotype in the HRS accounting for the effects of age, sex and the first 10 PCs.

Supplementary Table 9. Proportion of variants in the UKB height GWAS data removed by DENTIST using out-of-sample references.

	**MAF > 0.01**	**MAF > 0.001**
**UKBv8k**	0.04%	0.04%
**UKBv20k**	0.03%	0.11%
**ARIC**	0.33%	1.4%
**HRS**	0.34%	1.3%
**UK10K-WGS**	0.16%	0.73%

Supplementary Table 10. Proportion of variants removed in DENTIST analyses of the 10 published GWAS summary data sets using different LD references.

Trait	LD reference		
	**ARIC**	**HRS**	**UKBv3.8K**
BMI	**0.07%**	**0.05%**	**0.05%**
Height	0.67%	0.62%	0.75%
SCZ	0.13%	0.17%	0.63%
EA	0.21%	0.23%	0.22%
CAD	**0.86%**	**0.92%**	**1.46%**
T2D	0.23%	0.24%	0.22%
CD	0.30%	0.33%	0.66%
MDD	0.57%	0.59%	0.72%
BC	0.15%	0.17%	0.21%
OC	0.09%	0.10%	0.09%

Supplementary Table 11. Number of COJO signals from the COJO analysis of the 10 published GWAS data sets with and without DENTIST-based QC.

**Trait**	**DENTIST QC**	**LD reference for DENTIST and COJO**		
		**ARIC**	**HRS**	**UKBv3.8K**
BMI	No	728	744	727
	Yes	726 (-0.3%)	734 (-1.3%)	731 (+0.6%)
Height	No	3,145	3,109	3,040
	Yes	3,088 (-1.8%)	3,046 (-2.0%)	2,990 (-1.6%)
SCZ	No	155	150	142
	Yes	148 (-4.5%)	145 (-3.3%)	141 (-0.7%)
EA	No	480	485	467
	Yes	480 (-0.0%)	479 (-1.2%)	466 (-0.2%)
CAD	No	192	199	189
	Yes	**168 (-12.5%)**	**178 (-10.6%)**	**170 (-10.1%)**
T2D	No	274	263	267
	Yes	268 (-2.2%)	262 (-0.4%)	268 (0.4%)
CD	No	64	66	63
	Yes	61 (-4.7%)	63 (-4.5%)	62 (-1.6%)
MDD	No	45	45	44
	Yes	**45 (0%)**	**45 (0%)**	**42 (-4.5%)**
BC	No	194	193	181
	Yes	182 (-6.2%)	181 (-6.2%)	175 (-3.3%)
OC	No	12	12	12
	Yes	**12 (0%)**	**12 (0%)**	**12 (0%)**

The numbers in parentheses are the percentages of decrease (-) or increase (+) in the number of COJO signals after DENTIST-QC.

Supplementary Table 12. Estimates from LDSC analyses of the UKB height GWAS summary data with and without DENTIST-based QC.

	Number of variants	One-step approach		Two-step approach	
		h^2_{SNP}	Intercept	h^2_{SNP}	Intercept
**Reference = the discovery GWAS sample**					
**Benchmark**	1,114,780	0.46 (0.023)	1.13 (0.049)	0.41(0.017)	1.34 (0.030)
**Reference = HRS**					
**Without DENTIST**	1,118,873	0.45 (0.023)	1.27 (0.049)	0.42 (0.018)	1.37 (0.029)
**With DENTIST**	1,116,249	0.47 (0.023)	1.18 (0.043)	0.41 (0.017)	1.37 (0.029)
**Reference = ARIC**					
**Without DENTIST**	1,105,232	0.47 (0.025)	1.15 (0.047)	0.42 (0.018)	1.34 (0.030)
**With DENTIST**	1,103,783	0.47 (0.024)	1.14 (0.047)	0.41 (0.018)	1.34 (0.030)
**Reference = UKB-8K-1KGP**					
**Without DENTIST**	1,117,880	0.46(0.024)	1.15(0.049)	0.41 (0.018)	1.34 (0.031)
**With DENTIST**	1,117,453	0.46(0.023)	1.14(0.048)	0.40 (0.017)	1.34 (0.031)
**Reference = UK10K-WGS**					
**Without DENTIST**	1,074,399	0.47 (0.024)	1.13(0.049)	0.42 (0.018)	1.33 (0.030)
**With DENTIST**	1,092,923	0.47 (0.023)	1.11 (0.044)	0.41 (0.017)	1.33(0.030)
**Reference = 1KGP-EUR**					
**Without DENTIST**	1,133,151	0.48 (0.024)	1.15 (0.047)	0.43 (0.018)	1.33 (0.028)
**With DENTIST**	1,124,951	0.471 (0.023)	1.15 (0.048)	0.42 (0.018)	1.32 (0.023)

Standard errors are given in the parentheses.

Supplementary Table 13. LDSC results for the 10 published GWAS summary data sets with or without DENTIST-based QC.

Trait	DENTIST QC	LD reference for DENTIST and LDSC		
		ARIC	UK10K-WGS	UKBv3-8K
BMI	No	m=969,753;<br>h^2_{SNP}=0.172(0.006)<br>intercept=1.034(0.026)	m=955,844;<br>h^2_{SNP}=0.173(0.006)<br>intercept=1.008(0.025)	m=976,507;<br>h^2_{SNP}= 0.168 (0.006)<br>intercept= 1.026 (0.024)
	Yes	m=968,979;<br>h^2_{SNP}=0.170(0.005)<br>intercept=1.034(0.024)	m=955,229;<br>h^2_{SNP}=0.172(0.006)<br>intercept=1.009(0.025)	m=975,871;<br>h^2_{SNP}=0.164 (0.005)<br>intercept= 1.030 (0.024)
Height	No	m=965,367;<br>h^2_{SNP}=0.511(0.026)<br>intercept=1.368(0.124)	m=951,213;<br>h^2_{SNP}=0.510(0.026)<br>intercept=1.306(0.123)	m=971,821;<br>h^2_{SNP}=0.496(0.025)<br>intercept=1.347(0.122)
	Yes	m=958,942;<br>h^2_{SNP}=0.472(0.024)<br>intercept=1.275(0.008)	m=943,562;<br>h^2_{SNP}=0.46(0.021)<br>intercept=1.205(0.080)	m=964,237;<br>h^2_{SNP}=0.447(0.020)<br>intercept=1.234(0.076)
SCZ	No	m=1,121,978;<br>h^2_{SNP}=0.658(0.027)<br>intercept=1.036(0.013)	m=1,107,162;<br>h^2_{SNP}=0.651(0.025)<br>intercept=1.032(0.013)	m=1,092,944;<br>h^2_{SNP}=0.632 (0.025)<br>intercept= 1.038 (0.014)
	Yes	m=1,120,942;<br>h^2_{SNP}=0.656(0.027)<br>intercept=1.036(0.013)	m=1,099,585;<br>h^2_{SNP}=0.646(0.023)<br>intercept=1.032(0.013)	m=1,089,005;<br>h^2_{SNP}=0.628 (0.023)<br>intercept=1.039 (0.013)
EA	No	m=1138090;<br>h^2_{SNP}=0.113(0.003)<br>intercept=0.961(0.016)	m=1,110,625;<br>h^2_{SNP}=0.114(0.003)<br>intercept=0.935(0.016)	m=1,097,272;<br>h^2_{SNP}= 0.110 (0.003)<br>intercept= 0.948 (0.017)
	Yes	m=1,136,715;<br>h^2_{SNP}=0.113(0.003)<br>intercept=0.960(0.016)	m=1,106,705;<br>h^2_{SNP}=0.113(0.003)<br>intercept=0.932(0.016)	m=1,095,657;<br>h^2_{SNP}=0.11 (0.003)<br>intercept= 0.949(0.017)
CAD	No	m=1,147,517<br>h^2_{SNP}=0.060(0.004)<br>intercept=0.975(0.017)	m=1,131,860<br>h^2_{SNP}=0.060(0.004)<br>intercept=0.971(0.015)	m=1,115,519;<br>h^2_{SNP}=0.053 (0.004)<br>intercept=0.975 (0.016)
	Yes	m=1,124,598;<br>h^2_{SNP}=0.054(0.003)<br>intercept=0.977(0.016)	m=1,091,284;<br>h^2_{SNP}=0.054(0.003)<br>intercept=0.967(0.013)	m=1,085,336<br>h^2_{SNP}=0.053 (0.003)<br>intercept=0.976 (0.014)
T2D	No	m=1,103,665;<br>h^2_{SNP}=0.193(0.009)<br>intercept=1.077(0.028)	m=1,090,573;<br>h^2_{SNP}=0.192(0.009)<br>intercept=1.071(0.028)	m=1,115,665;<br>h^2_{SNP}=0.186(0.009)<br>intercept=1.080(0.029)
	Yes	m=1,101,989;<br>h^2_{SNP}=0.191(0.009)<br>intercept=1.070(0.025)	m=1,087,387;<br>h^2_{SNP}=0.190(0.009)<br>intercept=1.065(0.026)	m=1,113,978;<br>h^2_{SNP}=0.186(0.009)<br>intercept=1.070(0.025)
CD	No	m=1,148,525;<br>h^2_{SNP}=0.499(0.058)<br>intercept=1.023(0.011)	m=1,132,622;<br>h^2_{SNP}=0.501(0.057)<br>intercept=1.020(0.011)	m=1,115,890;<br>h^2_{SNP}=0.483 (0.058)<br>intercept=1.022 (0.011)
	Yes	m=1,147,392;<br>h^2_{SNP}=0.493(0.057)<br>intercept=1.022(0.011)	m=1,124,587;<br>h^2_{SNP}=0.503(0.056)<br>intercept=1.015(0.011)	m=1,112,341;<br>h^2_{SNP}=0.478 (0.057)<br>intercept=1.022 (0.011)
MDD	No	m=1,146,225;<br>h^2_{SNP}=0.059(0.003)<br>intercept=1.002(0.010)	m=1,130,372;<br>h^2_{SNP}=0.058(0.002)<br>intercept=0.998(0.010)	m=1,142,410;<br>h^2_{SNP}=0.056(0.002)<br>intercept=1.005(0.010)
	Yes	m=1,142,980;<br>h^2_{SNP}=0.059(0.002)<br>intercept=1.001(0.010)	m=1,120,468;<br>h^2_{SNP}=0.059(0.002)<br>intercept=0.993(0.010)	m=1,108,913;<br>h^2_{SNP}=0.056 (0.002)<br>intercept=1.003 (0.010)
BC	No	m=1,086,749;<br>h^2_{SNP}=0.129(0.012)<br>intercept=1.097(0.026)	m=1,106,705;<br>h^2_{SNP}=0.113(0.003)<br>intercept=0.932(0.016)	m=1,057,315;<br>h^2_{SNP}=0.1228 (0.012)<br>intercept=1.107 (0.030)
	Yes	m=1,085,928;<br>h^2_{SNP}=0.128(0.011)<br>intercept=1.091(0.023)	m=1,110,625;<br>h^2_{SNP}=0.113(0.003)<br>intercept=0.935(0.0163)	m=1,055,726;<br>h^2_{SNP}=0.123 (0.011)<br>intercept=1.086 (0.022)
OC	No	m=1,068,884;<br>h^2_{SNP}=0.050(0.010)<br>intercept=1.015(0.007)	m=1,054,645;<br>h^2_{SNP}=0.049(0.010)<br>intercept=1.016(0.007)	m=1,040,816;<br>h^2_{SNP}=0.047 (0.009)<br>intercept=1.018 (0.008)
	Yes	m=1,068,024;<br>h^2_{SNP}=0.050(0.010)<br>intercept=1.015(0.007)	m=1,052,337;<br>h^2_{SNP}=0.050(0.009)<br>intercept=1.014(0.007)	m=1,039,763;<br>h^2_{SNP}=0.047(0.009)<br>intercept=1.017(0.008)

m: the number of variants; h_{SNP}^2: the SNP-based heritability estimate; intercept: LD score regression intercept. Values in parentheses are the stand errors of the corresponding estimates.

Supplementary Table 14. Computing resources used by DENTIST in the analyses of the UKB height data on chromosome 2.

LD reference	Number of variants	Reference sample size	Memory usage	Number of CPUs	CPU time
**UKB.v3-20K**	626K	20,000	<6G	4	4h24min
**UKB.v3-20K**	95K	20,000	<6G	4	11min
**UKB.v3-8K**	626K	8,000	<6G	4	2h17min
**UKB.v3-8K**	95K	8,000	<6G	4	6min

Supplementary Notes

Supplementary Note 1. The exact and approximate models for z-statistic prediction

The method derived in the main text is under the null hypothesis of no association. Here we show a more general form of the derivation without this assumption. Following the definitions and notations in the main text, the exact form of the distribution of the z-statistic of a variant *i* in S1 conditioning on the variants in S2 is[1,2]

$$Z_i|\mathbf{Z}_{\boldsymbol{t}} = \mathbf{z}_{\boldsymbol{t}} \sim N(\boldsymbol{\Sigma}_{it}\boldsymbol{\Sigma}_{\boldsymbol{tt}}^{-1}\mathbf{z}_{\boldsymbol{t}} + (\mu_i - \boldsymbol{\Sigma}_{it}\boldsymbol{\Sigma}_{\boldsymbol{tt}}^{-1}\boldsymbol{\mu}_{\boldsymbol{t}}), \Sigma_{ii} - \boldsymbol{\Sigma}_{it}\boldsymbol{\Sigma}_{\boldsymbol{tt}}^{-1}\boldsymbol{\Sigma}_{it}')$$

where $\mu_i = E(Z_i)$, $\boldsymbol{\mu}_{\boldsymbol{t}} = E(\mathbf{Z}_{\boldsymbol{t}})$, and all the other notations are the same as those in the main text. Compared to Equation 1 in the main text derived under the null model, this equation has an additional term $\mu_i - \boldsymbol{\Sigma}_{it}\boldsymbol{\Sigma}_{\boldsymbol{tt}}^{-1}\boldsymbol{\mu}_{\boldsymbol{t}}$, which is zero under the null hypothesis of no association. Since $\boldsymbol{\Sigma}_{it}\boldsymbol{\Sigma}_{\boldsymbol{tt}}^{-1}\boldsymbol{\mu}_{\boldsymbol{t}}$ can be regarded as a predictor of μ_i, this term is expected to be small even under the alternative hypothesis, in line with our numerical analysis that under the alternative, the z-statistics predicted based on the approximation method are highly consistent with those predicted based on the exact method (**Supplementary Figure 15**).

Supplementary Note 2. Acknowledgments

UKB: UK Biobank was established by the Wellcome Trust medical charity, Medical Research Council, Department of Health, Scottish Government and the Northwest Regional Development Agency. It has also had funding from the Welsh Assembly Government, British Heart Foundation and Diabetes UK.

UK10K: The UK10K project was funded by the Wellcome Trust award WT091310. Twins UK (TUK): TUK was funded by the Wellcome Trust and ENGAGE project grant agreement HEALTH-F4-2007–201413. The study also receives support from the Department of Health via the National Institute for Health Research (NIHR)-funded BioResource, Clinical Research Facility and Biomedical Research Centre based at Guy's and St. Thomas' NHS Foundation Trust in partnership with King's College London. Dr Spector is an NIHR senior Investigator and ERC Senior Researcher. Funding for the project was also provided by the British Heart Foundation grant PG/12/38/29615 (Dr Jamshidi). A full list of the investigators who contributed to the UK10K sequencing is available from http://www.UK10K.org.

HRS: HRS is supported by the National Institute on Aging (NIA U01AG009740). The genotyping was funded separately by the National Institute on Aging (RC2 AG036495, RC4 AG039029). Genotyping was conducted by the NIH Center for Inherited Disease Research (CIDR) at Johns

Hopkins University. Genotyping quality control and final preparation of the data were performed by the Genetics Coordinating Center at the University of Washington.

ARIC: The Atherosclerosis Risk in Communities Study is carried out as a collaborative study supported by National Heart, Lung, and Blood Institute contracts (HHSN268201100005C, HHSN268201100006C, HHSN268201100007C, HHSN268201100008C, HHSN268201100009C, HHSN268201100010C, HHSN268201100011C, and HHSN268201100012C), R01HL087641, R01HL59367 and R01HL086694; National Human Genome Research Institute contract U01HG004402; and National Institutes of Health contract HHSN268200625226C. The authors thank the staff and participants of the ARIC study for their important contributions. Infrastructure was partly supported by Grant Number UL1RR025005, a component of the National Institutes of Health and NIH Roadmap for Medical Research.

Supplementary References

1. Lee, D., Bigdeli, T.B., Riley, B.P., Fanous, A.H. & Bacanu, S.A. DIST: direct imputation of summary statistics for unmeasured SNPs. *Bioinformatics* **29**, 2925-7 (2013).
2. Pasaniuc, B. *et al.* Fast and accurate imputation of summary statistics enhances evidence of functional enrichment. *Bioinformatics* **30**, 2906-14 (2014).
