# Supplementary material for: Improved analyses of GWAS summary statistics by reducing data heterogeneity and errors
Source: Nat Commun. 2021 Dec 8;12:7117. doi: 10.1038/s41467-021-27438-7 (PMC8654883; doi:10.1038/s41467-021-27438-7)
Supplement: Supplementary file 3 — Reporting summary [file 41467_2021_27438_MOESM3_ESM.pdf]

nature research

Corresponding author(s): Jian Yang
Last updated by author(s): Nov 17, 2021

Reporting Summary

Nature Research wishes to improve the reproducibility of the work that we publish. This form provides structure for consistency and transparency in reporting. For further information on Nature Research policies, see our Editorial Policies and the Editorial Policy Checklist.

Statistics

For all statistical analyses, confirm that the following items are present in the figure legend, table legend, main text, or Methods section.

n/a	Confirmed	
☐	☒	The exact sample size (*n*) for each experimental group/condition, given as a discrete number and unit of measurement
☐	☒	A statement on whether measurements were taken from distinct samples or whether the same sample was measured repeatedly
☐	☒	The statistical test(s) used AND whether they are one- or two-sided<br>*Only common tests should be described solely by name; describe more complex techniques in the Methods section.*
☐	☒	A description of all covariates tested
☐	☒	A description of any assumptions or corrections, such as tests of normality and adjustment for multiple comparisons
☐	☒	A full description of the statistical parameters including central tendency (e.g. means) or other basic estimates (e.g. regression coefficient) AND variation (e.g. standard deviation) or associated estimates of uncertainty (e.g. confidence intervals)
☐	☒	For null hypothesis testing, the test statistic (e.g. *F*, *t*, *r*) with confidence intervals, effect sizes, degrees of freedom and *P* value noted<br>*Give P values as exact values whenever suitable.*
☒	☐	For Bayesian analysis, information on the choice of priors and Markov chain Monte Carlo settings
☒	☐	For hierarchical and complex designs, identification of the appropriate level for tests and full reporting of outcomes
☐	☒	Estimates of effect sizes (e.g. Cohen's *d*, Pearson's *r*), indicating how they were calculated

Our web collection on statistics for biologists contains articles on many of the points above.

Software and code

Policy information about availability of computer code

Data collection	No data-collection software was used.
Data analysis	We implemented our method in a software tool called DENTIST. The source code is freely available at https://github.com/Yves-CHEN/DENTIST. We also used PLINK v1.90b3.38 for quality controls of the individual-level genotype data, GCTA v1.92.3 for simulation of quantitative traits based on real genotypes, GWAS analyses and COJO analyses, FINEMAP v1.4 for fine-mapping analysis, and LDSC v1.0.0 for SNP-based heritability estimation.

For manuscripts utilizing custom algorithms or software that are central to the research but not yet described in published literature, software must be made available to editors and reviewers. We strongly encourage code deposition in a community repository (e.g. GitHub). See the Nature Research guidelines for submitting code & software for further information.

Data

Policy information about availability of data

All manuscripts must include a data availability statement. This statement should provide the following information, where applicable:

- Accession codes, unique identifiers, or web links for publicly available datasets
- A list of figures that have associated raw data
- A description of any restrictions on data availability

All the data sets used in this study are available in the public domain. The UKB data are available through the UK Biobank Access Management System (https://www.ukbiobank.ac.uk/). The HRS and ARIC data are available in the dbGaP database under accession numbers phs000428 [https://www.ncbi.nlm.nih.gov/projects/gap/cgi-bin/study.cgi?study_id=phs000428] and phs000280 [https://www.ncbi.nlm.nih.gov/projects/gap/cgi-bin/study.cgi?study_id=phs000280], respectively. The UK10K data are available in the EGA database under accession numbers EGAS00001000108 [https://ega-archive.org/studies/EGAS00001000108] and EGAS00001000090 [https://ega-archive.org/studies/EGAS00001000090]. The 1KGP data are available at https://www.internationalgenome.org. The GWAS

summary statistics are available with unrestricted access through the links provided in the corresponding publications listed in Table 4. Source data are provided with this paper.

Field-specific reporting

Please select the one below that is the best fit for your research. If you are not sure, read the appropriate sections before making your selection.

[x] Life sciences [ ] Behavioural & social sciences [ ] Ecological, evolutionary & environmental sciences

For a reference copy of the document with all sections, see nature.com/documents/nr-reporting-summary-flat.pdf

Life sciences study design

All studies must disclose on these points even when the disclosure is negative.

Sample size	This study uses several cohorts as either the LD reference or GWAS discovery sample. The sample sizes of all the cohorts have been described in the paper. In the simulation study to demonstrate the power of DENTIST to detect errors, the sample size was 3,642, which was sufficient as demonstrated by the power illustrated in Figure 1c. In the simulation study to demonstrate the ability of DENTIST to improve COJO, given a sample size of 3,642, we had >99% power at p < 5e-8 to detect a simulated causal variant explaining 2% of the phenotypic variance. In the real analyses, given a sample size of 328,577, we had >99% power at p < 5e-8 to detect a genetic variant explaining only 0.03% of the phenotypic variance.
Data exclusions	Our study was restricted to GWAS data of genetically unrelated individuals of European ancestry (estimated genetic relatedness < 0.05). Standard QC was applied to the genotype data to remove genetic variants with a minor allele count < 5, Hardy-Weinberg equilibrium test P-value < 1e-6, genotype missingness rate > 5%, or imputation info score < 0.3. Additional QCs were performed in some analyses including the MAF threshold of either 0.01 or 0.001 for COJO analyses.
Replication	We repeated the simulation with each specific setting multiple times to assess the false positive rate and power of a statistical method. All the simulation replications were successfully performed.
Randomization	NA. We did not use any study design that required randonmization.
Blinding	NA. We did not use any study design that required blinding.

Reporting for specific materials, systems and methods

We require information from authors about some types of materials, experimental systems and methods used in many studies. Here, indicate whether each material, system or method listed is relevant to your study. If you are not sure if a list item applies to your research, read the appropriate section before selecting a response.

Materials & experimental systems

n/a	Involved in the study	
[x]	[ ]	Antibodies
[x]	[ ]	Eukaryotic cell lines
[x]	[ ]	Palaeontology and archaeology
[x]	[ ]	Animals and other organisms
[ ]	[x]	Human research participants
[x]	[ ]	Clinical data
[x]	[ ]	Dual use research of concern

Methods

n/a	Involved in the study	
[x]	[ ]	ChIP-seq
[x]	[ ]	Flow cytometry
[x]	[ ]	MRI-based neuroimaging

Human research participants

Policy information about studies involving human research participants

Population characteristics	This study makes use of data from the UK Biobank (project ID: 12514), HRS (dbGaP accession: phs000428), 1000 Genome Project, ARIC (dbGaP accession: phs000280.v6.p1) and UK10K (EGA accessions: EGAS00001000108 and EGAS00001000090). The UK Biobank (aged 40-69 with ~54% women) and UK10K (aged 17-85 with ~77% women) studies recruited volunteer participants in the UK. The HRS (aged 50 and older with ~59% women) and ARIC (aged 45-64 with ~56% women) participants were recruited in the US. The DNA samples were genotyped by either SNP array or whole genome sequencing technology. All the analyses in this study included only the individuals of European ancestry from these cohorts.
Recruitment	The participants in the UK Biobank have an age range between 40 and 69, genotyped on a customized Affymetrix Axiom array. HRS is a cohort recruited in the US with all the participants over the age of 50, who were genotyped using Illumina Human Omni2.5-Quad (Omni2.5) BeadChip arrays. ARIC is a cohort also recruited in the US with the participants aged from 45 to 64 from 4 communities (i.e., Forsyth County, NC; Jackson, MS; the northwestern suburbs of Minneapolis, MN; and Washington County, MD) and genotyped using Affymetrix 6.0 arrays. The UK10K data set consists of participants from the TwinsUK and ALSPAC cohorts recruited in the UK.
Ethics oversight	The UK Biobank has ethic approval from the North West Multi-centre Research Ethics Committee (MREC), which covers the UK. It also has approval in England and Wales from the Patient Information Advisory Group (PIAG) for gaining access to information that would allow it to invite people to participate. PIAG has since been replaced by the National Information Governance Board for Health & Social Care (NIGB). In Scotland, the UK Biobank has approval from the Community Health Index Advisory Group (CHIAG). This study is approved by the University of Queensland Human Research Ethics Committee (approval number: 2011001173) and the Westlake University Ethics Committee (approval number: 20200722YJ001).

Note that full information on the approval of the study protocol must also be provided in the manuscript.
